# Needs assessment for cultural adaptation of Strengthening Families Program (SFP 10-14-UK) in Brazil

**DOI:** 10.1186/s41155-018-0105-0

**Published:** 2018-09-25

**Authors:** Sheila Giardini Murta, Larissa de Almeida Nobre-Sandoval, Marina de Souza Pedralho, Thauana Nayara Gomes Tavares, Carlos Eduardo Paes Landim Ramos, Deborah Allen, Lindsey Coombes

**Affiliations:** 1 Department of Clinical Psychology, University of Brasília, Brasília, Brazil; 2 Faculty of Health and Life Sciences, Oxford Brookes University, Oxford, UK; 3 Departamento de Psicologia Clínica, Instituto de Psicologia, Universidade de Brasília, Campus Darcy Ribeiro, Brasília, DF CEP 70910-900 Brazil

**Keywords:** Prevention, Drug abuse, Parenting, Cultural adaptation

## Abstract

This study sought to evaluate the cultural adequacy of materials and procedures of the Strengthening Families Program (SFP 10-14-UK) and to identify requirements for its cultural adaptation to Brazilian families. The descriptive study had 33 informants, including external observers, managers, multipliers, facilitators, adolescents, and parents. The data were collected at a pilot application in the Federal District. Direct observation was applied to four intervention groups, with seven meetings of 150 min for families, parents/guardians and adolescents, and mixed nominal groups at the end of the interventions. The results, analyzed through content analysis and descriptive statistics, provided evidence that SFP was perceived as sufficiently appealing, culturally relevant, and partially clear. Recommendations for cultural adaptation of linguistic aspects of the materials and procedures were made, considering the cultural and educational differences of the participant families. Focus on implementation quality, including infrastructure, families’ mobilization and continuous planning, was recommended. Replication studies in other Brazilian regions and analyses of contextual and political dimensions are suggested.

## Background

Some of the main risk factors to Brazilian adolescents’ health are alcohol abuse, use of illicit drugs, risky sexual behavior, violence, and parental negligence (Brazilian Institute of Geography and Statistics, 2012). One suitable means of promoting health is the empowerment of adolescents, families, services, and communities through incentivization of proximal protective factors, such as family ties, and distal ones, such as access to integrated social policies focused on the advancement of opportunities for positive youth development (Garcia et al., 2014). With that in mind, in 2013, the Ministry of Health, through the Mental Health, Alcohol and Other Drugs coordination, adopted internationally developed evidence-based drug use prevention programs (Schneider et al., 2016; Medeiros et al., 2016; Pedroso et al., 2015), among which was the Strengthening Families Program (SFP) (Molgaard et al., 2007). This initiative aimed to respond to a demand for preventive actions planned in the Integrated Plans for Coping with Crack and other Drugs act (Brazil, Executive Order no. 7.179, May 20, 2010) and later in the program “Crack, Winning is Possible” (Brazil, Executive Order 7.637, December 8, 2011).

An international program was adopted due to the paucity of national programs for drug abuse prevention in the family environment that showed evidence of effectiveness and conditions for widespread use in public policies (Abreu & Murta 2016; Abreu et al., 2016). Advantages and disadvantages of using an international program have been raised. On the one hand, international programs may lack cultural sensitivity (Reese & Vera, 2007) and legitimacy (Damschroder et al., 2009), which can work against their local adoption by public managers and professionals in that field and demotivate the participation of families if they perceive the program as irrelevant to their context or unrelated to their experiences. On the other hand, the adoption of a program whose positive results are already known represents an impressive time savings, as the development, evaluation of efficacy, effectiveness, and the building of conditions for widespread use require a sequence of studies requiring significant time (Gottfredson et al., 2015)—more than that needed for the immediate implementation of public policies. Thus, the adoption of international programs necessitates studies to abet their cultural adaptation; at the same time, efforts must be collectively made by developmental agencies and researcher networks to expand the development, evaluation, and widespread use of nationally created and culturally appropriate programs for the prevention of drug abuse in the family environment.

The SFP was developed in the 1980s at the University of Utah, USA, by Kumpfer, DeMarsh, and Child (1989) as a selective prevention program and later revised by Molgaard, Spoth, and Redmond (2000) from the University of Iowa, USA, who assigned it a shorter format of universal prevention. It is based on the theory of family systems, social cognition theory, the model of resilience, and socio-ecological models. According to the last, family cohesion, parental supervision, and communication of positive values from the family constitute, in the family environment, the most relevant protective factors for prevention of drug abuse (Kumpfer et al., 2010). True to its theoretical foundations, the content of the Strengthening Families Program focuses on promoting parenting practices of emotional support and encouraging responsibility; on assertive social skills for coping with the peer pressure, skills for emotional regulation, and life projects for adolescents; and on strengthening family cohesion, family communication, values, and resources for problem solving.

A systematic review indicated SFP as effective in preventing alcohol misuse in the long term (over 3 years) (Foxcroft et al., 2003). Given the evidence for SFP’s effectiveness obtained from randomized clinical trials with follow-up evaluations in delaying age of first alcohol use (Spoth et al., 1999) and improving interpersonal mindfulness in parenting, quality of the parent-juvenile relationship, juvenile behavior management (Coatsworth et al., 2010; Coatsworth et al., 2015), school engagement, and academic success (Spoth et al., 2004), it has been adapted and implemented in several European countries (for example, Allen et al., 2007; Ortega et al., 2012; Skärstrand et al., 2008; Stolle et al., 2011) and in countries of low and middle income (Maalouf & Campello, 2014). The program adopted in Brazil is a version for children and adolescents aged 10 to 14, adapted in the United Kingdom (Strengthening Families Program-10-14-UK) (Allen et al., 2014), which had been initially named “Strengthening Families Program” by the Ministry of Health, but later changed to “Strong Families Program” (respectively “Programa Fortalecendo Famílias” and “Programa Famílias Fortes” in Portuguese). The original intervention plans included the participation of parents/guardians and adolescents in seven weekly sessions. After these, there were four monthly follow-up sessions, to take place between the 3rd and 12th months after the end of the seventh weekly session.

Once the SFP was adopted, its implementation was an intersectoral act, which included inviting the following for participation: The Ministry of Social Development and Fight Against Hunger (Department of Basic Social Protection), which joined the program in the beginning of 2014, and the Ministry of Justice (National Department of Drug Policies), which joined the program by the end of 2014, effectively participating in its implementation in the beginning of 2015 (Miranda, 2015). The implementation of culturally appropriate, science-based programs of drug abuse prevention that also include the participation of families is aligned with the guidelines of public policies for prevention of problematic drug use (http://www.justica.gov.br/sua-protecao/politicas-sobre-drogas/prevencao-e-tratamento/prevencao/prevencao). The starting point for the implementation of SFP was in the context of the Comprehensive Family Attention Program. This service integrates The National Policy of Social Assistance (Brazil, Ministry of Social Development and the Fight Against Hunger, 2004), offered in the Social Assistance Reference Centers, whose purpose is the provision of interaction spaces, the strengthening of family ties, the development of autonomy and empowerment, and access to social-assistance for socially and economically vulnerable families (Brazil, Ministry of Social Development and Fight Against Hunger, 2012). One of the Ministry of Health’s first steps after adopting the Strengthening Families Program in 2013 was to analyze its cultural adequacy for Brazil, the goal of the present study.

The adoption of family-based programs developed in other contexts and their transfer to a new culture must be done carefully respecting the cultural preferences and local resources to reach the targeted efficacy and effectiveness (Mejia et al., 2016). Cultural adaptation covers the systematic modification of an evidence-based intervention protocol that accounts for the culture, context, and language, making it compatible with the cultural values, beliefs, and norms of the target population or client while preserving fidelity to the core components of the program. The goal of the cultural adaptation of preventive programs in the healthcare field is the achievement of activities, procedures, and materials that are perceived as appealing, motivational, clear, understandable, suitable, and relevant for the new context (Castro et al., 2010). Thus, the adaptation of programs to the target population’s culture is one of the elements that ethically qualifies the process for implementation by respecting the values, needs, and interests of the participants (Reese & Vera, 2007). Such elements influence the responsiveness, compliance, and retention of the program’s participants, which in turn positively affect its mechanisms of impact, and finally, its effectiveness (Moore et al., 2013).

The SFP’s authors recommend that the process of cultural adaptation involve only its superficial structure, including the use of culturally grounded welcome activities, dancing, songs, photos, and stories, whereas the program content, duration, structure, and session order should be preserved. As such, the combination or exclusion of sessions, reorganization, change or suppression of components, and homework elimination are not recommended (Kumpfer et al., 2002). Due to its systematic character, a few authors (Kumpfer et al., 2012; Kumpfer et al., 2008) recommend that the cultural adaptation be preceded by a preliminary implementation—after the translation of guidelines and materials and formation of facilitators—where only minimal adaptations should be made in the procedures and materials with the purpose of testing these in a small sample for the cultural appropriateness of the program. With such an experience as a starting point, the next step is to identify the necessary adaptations and to generate inputs capable of guiding the future adaptation based on the directives given by the needs assessment.

With that purpose, in 2013, a preliminary implementation of SFP was done in the Federal District with the objective of applying the lessons from the experience for decision-making concerning the program’s cultural adaptation, to be done in the next stage as recommended by specialists (Kumpfer et al., 2012; Kumpfer et al., 2008). As such, this study was carried out with two objectives: first, to evaluate the cultural appropriateness of the program—that is, to which degree were the materials and activities of Strengthening Families Program perceived as appealing, clear, and suitable for the Brazilian culture—and second, to assess requirements for cultural adaptation guided by cultural relevance, appeal, and clarity of the procedures and materials.

## Methods

### Participants

The study design was descriptive, with qualitative and quantitative measures and multiple informants. There were 33 informants, comprising external observers (*n* = 4), who received SFP training from the researchers responsible for the English version of the Program adopted in Brazil; mothers (*n* = 4), fathers (*n* = 2), and adolescents (*n* = 7) who were participants in Strong Families Program; facilitators (*n* = 6) who implemented the Program’s sessions; federal multipliers (*n* = 6) who were trained to act as creators of new implementation teams; and public managers (*n* = 4) with ties to the Ministry of Health. The external observers were recruited from researchers at the University of Brasilia; the facilitators, mother, fathers, and adolescents were recruited from the Coexistence and Relationship Strengthening Services, the Social Assistance Reference Centers (both part of social policies targeting vulnerable families), and the Legal Guardianship Council (a service focused on the protection of the rights of children and adolescents); and the multipliers and public managers were recruited from the Ministry of Health team. All the participants of this study were properly informed about their rights and expressed their agreement to participate in this study via an informed consent form. They were told about the voluntary character of the participation, the objectives of the research, the possibility of desisting at any moment, the protection of their identities in the data analysis and publications, access to the study results, anticipated uses of the data, the minimal risk, and the potential benefits of the study.

While the external observers followed the four group sessions of the SFP in loco to judge its cultural adequacy, the remaining participants were divided into mixed nominal groups, taken through the program and then indicated items in need of enhancement for its cultural appropriateness. The methodological choice of the inclusion of different actors followed the recommendations in the literature specializing in cultural adaptation, which recommends a perspective both collaborative and participative (Ferrer-Wreder et al., 2012; Kumpfer et al., 2008; Burkhart, 2015).

### Strengthening Families Program

The SFP was applied to selected families among users of the Coexistence and Relationship Strengthening Services, the Social Assistance Reference Centers, and the Legal Guardianship Council. All told, the services have assisted a total of 93 parents or legal guardians and 107 adolescents. Only a subsample of the assisted people served as informants for this study, as described above. The parents/guardians and adolescents were assigned to eight intervention groups and the intervention took place between October and December of 2013 in six sub-districts of the Distrito Federal.

The program was carried out in seven sessions, each lasting approximately two and a half hours. The first part of the meeting was held in two separate groups, one for the parents/guardians (family) and the other for adolescents, both lasting approximately 60 min. The second was held jointly, i.e., with both the adolescents and their parents/guardians. The participants were offered refreshments at the beginning, end, or between the first and second parts of the meetings according to their wishes at the time. Six federal multipliers and 15 social educators from the Coexistence and Relationship Strengthening Services, the Social Assistance Reference Centers, and other professional entities associated with the Federal District acted as facilitators. These professionals held degrees in psychology, social work, and history. The federal multipliers were professionals selected by the Ministry of Health to facilitate the program and support the process of applying the intervention in the pilot phase as well as to form new implementation teams in the future phase of spreading the program through Brazil.

The content addressed in the meetings, provided in a manual translated beforehand by technicians of the Ministry of Health (Allen et al., 2014), encompassed parenting practices such as synchrony and encouraging responsibility, supporting the adolescents’ dreams, family values, appreciation of family members, assertive and empathetic communication, regulation of emotions, stress management, family leisure, friendship quality, peer resistance skills, and support and community services. All the sessions with the families included the use of audio and video material (DVD) presenting situations that promote discussion of the items planned in the program. Periods for discussion were timed and provided in the videos. With the videos as a starting point, the facilitators mediated discussions with the participants about acknowledgment and the learning of a new repertoire of behaviors that promote protective factors and decrease risk factors associated with violent behavior, alcohol abuse, and abuse of other substances among others.

To stimulate participation, for most of the sessions, transportation was offered for the families and caregivers provided to mind children less than 10 years of age and not participating in the program’s activities. Such services, as well as gifts to give them as presents over the meetings, are specified in the original intervention.

### Instruments and procedures for data collection

A field diary for use by the facilitators and external observers to evaluate the fidelity of the sessions’ implementation, the participants’ engagement, and the cultural appropriateness of the SFP was developed for this study. In this paper, only the data informed by the external observers will be reported. The instrument comprised of two parts. The analysis of the first part is out of this study’s scope. It consisted of a registry of the adopted procedures, the order in which they occurred, and a record of episodes of greater and lesser engagement of the participants in the activities. The second part, which is the object of analysis of the present study, consisted of a 5-point Likert for the external observers to rate the sessions’ procedures in terms of appeal (to what extent are the procedures interesting and promote engagement and motivation of the participants?), relevance (to what extent do the procedures relate with the local culture and contemplate relevant elements of the Brazilian culture?), and clarity (to what extent were the procedures carried out in a way that was understandable to the participants?). For low scores (1, 2, or 3), it was requested that the observer indicate what could be changed to improve the cultural appropriateness of the SFP for Brazil with open questions such as: “What must change to make the session more coherent for the local culture?”. At the end, the observer was requested to openly and honestly record impressions of the weak and strong points of the sessions.

Direct observation of the seven meetings carried out in the four groups of the SFP was done. During the first hour of the intervention, an observer dedicated himself to the observation of the group of adolescents, while a second observer observed the group of parents/guardians. In the second hour, both observers followed the joint meeting. The observers were instructed to adopt a non-invasive behavior during the sessions and to make notes in the field diary immediately after the joint meetings.

Based in a procedure developed by Allen et al., (2007), the Script for Nominal Groups with Multiple Actors was elaborated, comprised of guide items which addressed the used videos’ and activities’ cultural adequacy accordingly to the situation, approaches, language, examples, narration, and scenarios. These included (a) aspects of the videos and activities the closest and most relevant to and those most distant and different from Brazilian culture, (b) most and least appealing aspects of the videos and program activities, and (c) aspects that must be kept and that must be changed in the adaptation of the videos and activities as well as in the working of the program (for instance, the length and number of facilitators).

The Script for Nominal Groups with Multiple Actors was used 1 month after the end of SFP, when three mixed nominal group meetings were held, with the participation of parents/guardians, adolescents, facilitators, multipliers, and public managers to assess elements to be adapted in the program targeting its appeal and cultural relevance to Brazil. The groups represent four low-income regions of the Federal District where the SFP was carried out. Mixed nominal group 1 comprised 12 participants: two female parents/guardians, three adolescents (two boys and one girl), three facilitators, two multipliers, and two public managers connected the Ministry of Health. Mixed nominal group 2 comprised nine participants: two female parents/guardians, two male adolescents, two facilitators, two multipliers, and one public manager. Mixed nominal group 3 comprised eight participants: two parents/guardians, one adolescent, one facilitator, two multipliers, and two public managers.

The procedure described by Allen et al., (2007) was employed. An excerpt of the fifth session video was selected to stimulate the evaluation. The exhibited excerpt, 3 min in length, addressed what school must offer to the parents, followed by a scene between two parents playing pool and talking about what they need to know when their children leave the house. The participants were invited to discuss and rank, first individually, then consensually, aspects of the material or program activities to be adapted to become appropriate to Brazilian culture. The following instruction was given: “What do you judge should be improved in the material and in the program’s activities for it to be more like ‘our way in Brazil’?”, complementing with “What else do you believe must be done for the program to work well and succeed in Brazil?”. Finally, they were invited to rank some of the listed aspects (the five or ten most important), in priority order, for adaptations in the next edition of the program. From these rankings, as the final product of the nominal group, a list of items recommended for adaptation was generated. Written records of the group’s production while under observation were made.

### Data analysis procedures

**Fig. 1:**
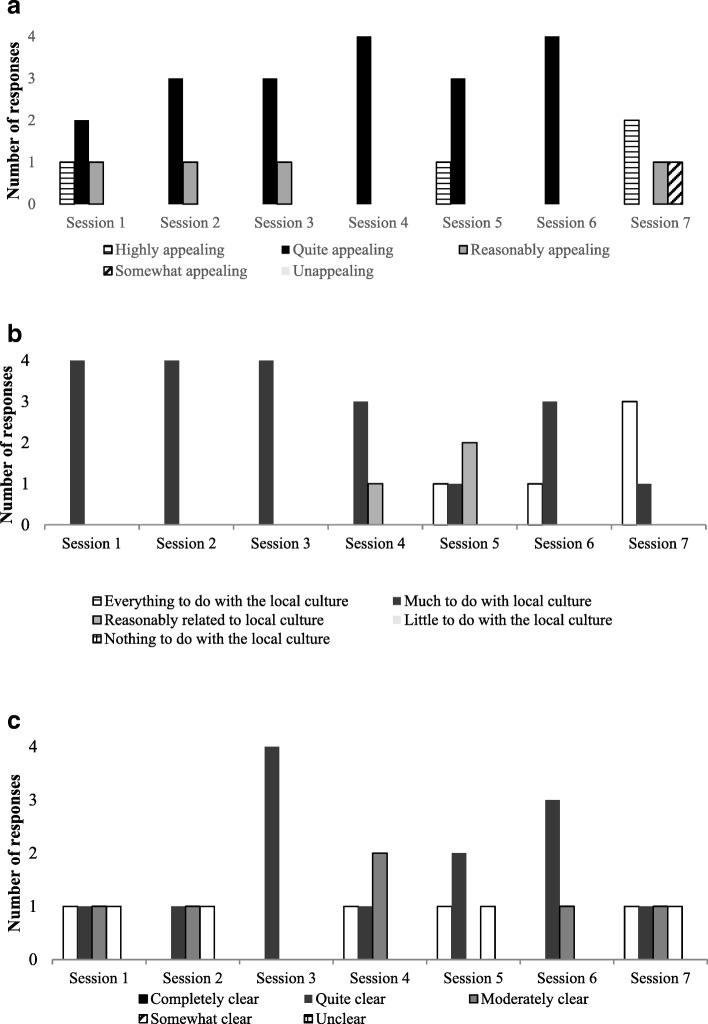
External observers’ perceptions. **a** Appeal of the procedures, **b** Cultural relevance of the procedures, and **c** Clarity of the procedures. (*N*: session 1 = 6 observations; session 2 = 5 observations; session 3 = 3 observations; session 4 = 4 observations; session 5 = 3 observations; session 6 = 4 observations; session 7 = 4 observations)

The quantitative data derived from the field diaries of the external observers’ judgments regarding clarity, relevance, and appeal of the meetings were analyzed with descriptive statistics (frequency) using the software SPSS v.20.0, and graphics were generated for visual inspection (Fig. 1). The qualitative data derived from the mixed nominal groups were analyzed by content analysis (Creswell, 2007). The transcriptions were submitted to repeated readings; categories and subcategories were identified and refined after a discussion among the authors. The needs identified for cultural adaptation of SFP were grouped in the following categories: (1) material and procedures, (2) logistics, (3) length, and (4) recruitment and mobilization. The elements comprising the categories are described in Table 1.

**Table 1:** Cultural adaptation recommendations according to the nominal groups of parents/guardians, adolescents, multipliers, facilitators, and managers

		Group 1	Group 2	Group 3
Materials and procedures	Enhance DVD quality	x	x	x
	Adapt the language to the target audience	x	x	x
	Reduce written activities or add personnel to support these activities	x	x	x
	Offer copies of the material to be taken home		x	
	Include in the activities and scenes themes based on the reality of Brazilian families and adolescents, such as sexuality, violence, and bullying/harassment		x	
	Adapt activities to the local culture			x
	Adapt the consequences of inappropriate behavior to the local culture		x	
Logistics	Separate child and adolescent participants by age range (10 to 13 years and 14 to 17 years)	x	x	
	Offer the program in the evening	x		
	Increase the number of caregivers for children less than 10 years of age	x		
	Always offer transportation to all meetings			x
	Offer a greater variety of snacks			x
	Invest in planning, from announcement to the execution of the sessions			x
	Limit the number of participants per group			x
Duration	Increase the time for debate	x	x	
	Spread the program across more sessions (9 or 10)	x	x	
Recruitment and mobilization	Enhance the recruitment of participants	x	x	x
	Present the SPF to families before the first session of the program			x
	Improve the promotion of the program			x

## Results

### Appeal, cultural relevance, and clarity of the Strong Families Program according to external observers

The cultural appropriateness of the procedures performed was evaluated by external observers according to three criteria: appeal, cultural relevance, and clarity. According to the external observers, the first sessions were quite appealing and reasonably appealing, the middle sessions were quite appealing, and the final sessions varied between somewhat appealing and highly appealing. No session was classified by the external observers as unappealing. According to Fig. 1, the majority of sessions were rated as quite appealing by the external observers.

As for the relevance of the procedures to the local culture, those adopted in the initial and middle sessions were considered to have “much to do with the local culture” by the observers. In the end sessions, cultural relevance varied between “reasonably related to local culture” and “everything to do with the local culture”. No session was evaluated as having “nothing to do with the local culture” or “little to do with the local culture” (see Fig. 1).

While the appeal and cultural relevance of the procedures were considered appropriate by the observers, with relative homogeneity between sessions, the clarity of the procedures varied greatly over the program’s implementation, ranging from insufficient clarity (unclear) to sufficient (completely clear). The unclear sessions, which are at greater risk of being misunderstood by the participants, occurred at the beginning, middle, and end of the program. Nonetheless, sessions were most commonly reported as having a proper degree of clarity, “quite clear,” or “completely clear,” as seen in Fig. 1.

Although the external observers offered specific suggestions for improving the appeal, cultural relevance, and clarity, the data will be presented as a set. These suggestions, presented with the intention of making SFP more motivating, culturally relevant, and understandable to the participants, fall along two lines: cultural appropriateness and implementation quality.

In the first category, adaptations to the language of the activities and materials of the SFP were suggested. Suggested language adaptations included changing the names of cars, games, objects, and places as well as typically English day-to-day activities for others common in Brazil: for example, replacing “family crest” for “family flag.” Other suggestions for activities included the use of strategies that do not require reading or writing, as a significant number of the adults were illiterate and younger children presented difficulty reading, and customizing the playful activities to the developmental necessities of the younger children (10) and older adolescents (14). Finally, examples of suggested adaptations for materials include improving the quality of dubbing and sound of the DVDs, including extended family—beyond grandparents—who might offer support in the branches of the “family tree” activity (session 2), thus taking into account multiple family configurations and adapting the activities that require reading, as the illiteracy of the parents/guardians affects the engagement and length of the activity, compromising fidelity.

In the second category, implementation quality, greater attention to planning, introduction, and conclusion of the activities was recommended, as well as to the facilitator’s ability, time management, and group management. Even though such recommendations are not directly related to cultural adaptation, the external observers considered them capable of impacting the understanding and appeal of the procedures for the participants. It was suggested that the sessions be planned together by the team of facilitators to advance everyone’s performance quality, improve the distribution of functions, and facilitate uniform understanding among facilitators, which should impact the clarity of the instructions given to the group. It was also suggested that the session’s material be prepared in advance, as too much time was spent writing information on the board, which allowed the participants to get distracted. To boost the understanding and meaning of the procedures, it was suggested that the objectives and relevance of the activities be explained and concluded, summarizing its main aspects and improving the transference of the reflections and lessons to day-to-day interactions, before moving on to the next planned activity. The external observers suggested the facilitators improve their communication abilities, such as using understandable language in place of technical jargon (such as “empowerment”), using examples, and giving the group feedback through empathetic verbalization and paraphrasing. Regarding time management, synchronizing the parent/guardian sessions and adolescents to end them at the same time and avoid delays in the family sessions, which might reduce the participants’ interest, was made. Finally, as for group management, boosting interaction between participants and the group’s affective closeness was suggested.

### Necessities for cultural adaptation of the Strong Families Program in Brazil according to adolescents, parents/legal guardians, facilitators, multipliers, and public managers

The necessities for adaptation ranked by the three mixed nominal groups, comprised of adolescents, parents/legal guardians, facilitators, multipliers, and public managers, are described in Table 1. All groups agreed upon the need to reduce written activities, enhance the quality of the DVDs, correct linguistic aspects, and improve recruitment of families. While the first three items relate to cultural adaptation directly, the last one is about implementation quality.

As the external observers proposed, the participants of the mixed nominal groups suggested reducing the written activities, given that many participants of SFP cannot read or do not read fluently, which can cause feelings of discomfort and alienation from the group, beyond prolonging the session, demotivating the participants, and damaging fidelity. Regarding the DVD quality, improvements were suggested regarding the actors (using Brazilian actors wearing local outfits), scenery (for example, mothers should meet in the street, sidewalk, bus stop, park, children’s park, and beauty salon; fathers, in turn, should meet at the house gate, soccer square, or at work), language (for example, using prosody, using Brazilian names, saying the first name instead of the surname, and using slang and less formal terms), content (for example, in activities discussing peer pressure, it was suggested that pressure to steal be replaced with pressure to “graffiti,” “take money to buy marijuana,” “skip class,” “gang up to bully your classmates,” and “steal candy from the supermarket”), and audio (for example, synchronization between speech and actions).

The necessity of making the language more relevant to the local Brazilian culture (using slang and local terms and expressions) and improving non-verbal aspects (such as the emotion expressed by the intonation) was indicated. Among the linguistic adjustments, the replacement of places, musical instruments, cars, and names with Brazilian names or nouns familiar to the local reality (for instance change “clarinet” for “acoustic guitar”) was suggested.

Finally, it was the groups’ consensus that the process of the announcement, recruitment, and mobilization of families must be improved. It was recommended that the strategies of the announcement and presentation of SFP to the families before the first session be broadened. Such measures were considered to have the potential for boosting the attraction and adherence of the families. Even though not directly focused on cultural adaptation, these were considered relevant for advancing the perception and appeal of SFP and motivating participation.

Two mixed nominal groups pointed to the need to offer distinct activities for children and older adolescents and to extend the duration of SFP to make time management more flexible and to allow more families the opportunity to participate. Other suggestions made by only one of the mixed nominal groups can be seen in Table 1.

Among the ideas proposed in Table 1, attention is drawn to the suggestion of making SFP’s material available to be taken home by parents and adolescents, which reveals the level of engagement of the families with the program. Parents/guardians expressed their desire to access the material from home for a consultation and to present the videos to other family members. Moreover, they conveyed their wishes to act as program multipliers or collaborators in its implementation (mixed nominal group 1 and 2). Diffusion of the program, that it be spread and expanded, was recommended: “it has to go far, to catch on in the schools...” (participant from mixed nominal group 2). Furthermore, it was reported that the tools and/or abilities discussed in the program are still used, such as leaving a paper note with a compliment, posting received materials on the refrigerator, checking the score table, and hanging the family tree on the wall (mixed nominal groups 1 and 2). The beneficial effect of the collective participation of family and adolescents was emphasized, as evidenced by following reports: “M. was very happy when his mother started to participate, and he was more affectionate”, “We notice that E. was also very happy when her father showed up, he keeps saying ‘this is my dad’”, (participant from mixed nominal group 2). Hence, even though there are different necessities of cultural adaptation, the perception of Strong Families Program as relevant, and whose continuance was desired, was evident in the three mixed nominal groups, as reported by the participants.

In the same vein, the facilitators reported that the execution of the SFP in the Coexistence and Relationship Strengthening Services improved the functioning of the service by offering a method of intervention coherent with the general objectives of the service (mixed nominal groups 1, 2, and 3), that is, the strengthening of family ties. According to their perception, it acts as a guideline for their efforts: “The program worked super well! It explored the work with families. I was able to carry out the work. Fantastic!” (participant of mixed nominal group 3). In summary, participants and facilitators reported a positive perception of SFP’s social validity, even though it needed further cultural adaptation and improvements in implementation quality.

## Discussion

This study’s main objective was to evaluate the cultural adequacy of the materials and activities of the Strengthening Families Program and to raise the necessities for its adaptation to Brazil. The results show a consensus among external observers, participants, implementation team, and management team regarding the need to perform adaptations in the superficial structure of SFP, including adaptations in the materials, activities, and language. Resembling findings of studies about SFP’s cultural adaptation carried out in other countries (Allen et al., 2007; Maalouf &Campello, 2014; Ortega et al., 2012; Stolle et al., 2011), except for Sweden (Skärstrand et al., 2008), adaptation needs for its deep structure, such as in values and risk factors and associated protective approaches, were not recommended. Such data indicates that the protective factors promoted by the Strengthening Families Program, as revealed in the objectives and content of the sessions, are relevant for the participating Brazilian families. Thus, the data suggests that the SFP was perceived as sufficiently appealing and culturally relevant from the perspective of the diverse actors participating in this study.

From the external observers’ point of view, the appeal and cultural relevance were more satisfactory than its clarity. It is possible that the reduced clarity may be attributed to at least, two conditions. The first was the very frequent use of procedures and materials that presuppose abilities of reading and writing, as assumed in the SFP’s guideline, which was incompatible with the characteristics of the target population assisted, especially the adults. The assisted families belong to a highly economically vulnerable class which has been historically deprived of access to education or at least to quality education. In Brazil, literacy and school performance compatible with the children’s age are still goals being pursued instead of consolidated victories (http://www.todospelaeducacao.org.br/biblioteca/1522/de-olho-nas-metas-2013-14/). As such, the cultural adaptation of SFP in Brazil must account for the adults’ illiteracy and the functional illiteracy of younger children. As discussed by Burkhart (2015), the cultural adaptation of international evidence-based programs must go beyond language appropriateness, values, beliefs, and meanings to include adaptation to the social, economic, and political context of the target country. Thus, it seems fundamental that the management teams account for the educational and social-economic context in which the participating Brazilian families live or risk not achieving the final goals of the SFP.

The second condition that may explain the lack of clarity perceived in the procedures’ execution may be related to gaps in the facilitators’ performance. It may have been caused by problems in sessions’ execution, as indicated in mixed nominal group 3 (Table 1). In it, the lack of clarity identified by the external observers loomed largest when presenting the activities and concluding them in a way that spelled out its objectives, meanings, and connections with day-to-day living, thus making them easier to understand and learn effectively. In this sense, it is possible to speculate that the low clarity may have damaged the delivery of essential elements of the intervention, thus decreasing implementation fidelity. One could ask if the lack of ability to clarify the meaning of procedures and to manage groups would unveil deficits in the earlier stages, in prior training to facilitate SFP beyond the session planning. It is possible that the selection of professionals for training and the training’s length, content, or delivery require some enhancement. Improvements in the pre-implementation stage with adjustments in facilitator selection and training could be tested and their relation to implementation fidelity examined in future studies.

The observers and mixed nominal group participants were in agreement regarding the perceived necessity of paying attention to the implementation quality, which in turn affects the perception of the children, adolescents, and parents/guardians of the clarity and appeal of the procedures. The recruitment, mobilization, and selection of families were emphasized, including investments in planning means to announce Strong Families Program, to attract families, and to select and then retain them over the course of the sessions. One suggestion was to present the program ahead of session 1, to present SFP to the families to motivate them to participate. This sort of measure might also facilitate the selection of families whose profile is compatible with that anticipated for participation in SFP. Families who are in positions of severe deprivation should be assisted in other services. The insertion of such families in the groups demands other strategies of intervention and affects the group process, possibly causing demotivation and hopelessness among the participants. These findings are in line with those of Segrott et al. (2017), which also pointed out a challenge to recruit families for the SFP in Wales, UK. Therefore, investments in the recruitment, selection, and preparation of the families for inclusion in the program should be a priority.

Among the suggestions proposed by the mixed nominal groups, attention to the support infrastructure, such as caretakers for younger children, snacks, and transportation, stands out. It is known that institutional support and local infrastructure comprise variables that affect the sustainability of health programs (Schell et al., 2013) and, in SFP’s implementation, the said infrastructure is a condition for SFP’s implementation. On the other hand, such elements may make the offering of SFP more expensive and complex, which may compete with its adoption, large-scale implementation, and sustainability, as already verified in European countries (Burkhart, 2015). The present author, when analyzing the European experiences when implementing Strengthening Families 10-14, argues that, in different countries, the infrastructure and services of the locale that received the program had an impact on its sustainability. In Brazil, the program’s implementation becomes even more complex due to its intersectoral execution (Miranda, 2015). The preparation and articulation of the local services that will receive the program have a direct impact on its success. Thus, the expansion of the program must be carefully followed and its feasibility examined under the light of cultural, political, economic, and organizational factors.

Indeed, the major problem with taking an evidence-based program from the international area and implementing it in a new country is how to keep its evidence intact in light of the new contextual demands. The context is dynamic and complex and includes cultural, political, economic, epidemiological, geographic, ethical, and legal dimensions (Pfandenhauer et al., 2017). Depending on the demands from the adopting community (e.g., families, providers, community leaders, political leaders), delivery system (e.g., organizational capacity for implementation), and support system (e.g., availability of technical assistance and training), the program may be substantially modified to better suit the local context (Durlak and DuPree, 2008). A lack of consideration of such contextual requirements may result in a program with poor fit to the real world, damaging its ownership by the community and sustainability (Kemp, 2016). In doing such adaptations, the program’s core elements and targeted protective process should be preserved during the implementation process. To pursue these two goals, contextual adaptation and fidelity, a theoretically, culturally, and politically competent leadership and work team are necessary.

Despite the implementation challenges, the national dissemination of Strong Families Program may represent an unprecedented advance in social policies for families in situations of poverty and in the policies of prevention of risks to and promotion of mental health in Brazil. This would favor the mental health expansion of the care network, as has been requested by several sectors of society (United Healthcare System, National Health Counsel, Organizing Committee of the Fourth National Conference on Mental Health – Intersectoral, 2010). Such an advance would be even more prominent, particularly in the prevention of drug abuse, having as reference the former initiatives implemented to prevent the misuse of drugs in Brazil (Casela et al., 2014).

If, on the one hand, the adoption of international evidence-based drug prevention programs represents an advance over the current situation, on the other hand, the investment and evaluation of programs produced nationally must be considered at the same time. It is urgent to apply resources to stimulate researchers concerning the development, evaluation, and diffusion of evidence-based drug abuse prevention programs. Such an investment would allow the strengthening of integrated social policies (Garcia et al., 2014) focused on protective factors both familial and educational, as well as communitarian, economic, and cultural.

This study presents limitations. The first one is external validity. The data obtained in this study result from applying SFP in the Federal District alone. Given the economic disparities among Brazil’s regions and its cultural and geographic diversity, the same level of appeal, cultural relevance, and clarity of SFP found in this study cannot be assured and replicated for other regions. The second limitation is related to the validity of the observational data, which was limited by the absence of inter-coder reliability among observers. Unfortunately, while it was possible to have two external observers in the family sessions, the separate parent/guardian and adolescent sessions had only one each. As a methodological strategy, to decrease the observer’s intrusive effect, it was decided not to video record the sessions, which prevented further analysis by other observers. This limitation is mitigated by the different strategies of data collection used, which made it possible to compare the observations with the verbal data from mixed nominal groups, and consistency between them was examined and found.

In conclusion, the SFP presented as relevant to the Brazilian culture, with good levels of appeal and cultural relevance. Given such findings, it is recommended that it be expanded to other regions of the country, accompanied by new evaluations of cultural adequacy as well as effectiveness. Effectiveness studies could benefit from using realistic evaluation, a theory-based approach to evaluation, to shed light on the impact of SFP in Brazil and its complex chain of change by combining qualitative and quantitative methods to examine patterns of outcomes and the mechanisms and contexts that favor them (Salter &Kothari, 2014). Furthermore, effectiveness evaluation of a culturally adapted version of SFP to Brazilian vulnerable families might increase evidence under Brazilian conditions and broaden the still scarce local evidence of preventive programs (Abreu &Murta, 2016; Abreu et al., 2016).

Cultural adaptation is a continuing process. Thus, the large-scale implementation of the SFP in Brazil may reveal new cultural adaptation necessities. This is especially relevant for communities and regions distinct from that in which the data of the present study was collected, such as rural communities and small cities from other Brazilian states. Additionally, performing new studies aimed at SFP’s cultural appropriateness in other Brazilian regions would result in inputs for cultural adaptations carried out in a collaborative manner. It is the consensus among the scholars in the field that cultural adaptation be performed not by one actor only (researchers, for instance), but with the participation of all the involved in the implementation process, along with key informants of the target community and members of the management of the service to adopt the intervention (Ferrer-Wreder et al., 2012; Kumpfer et al., 2008; Burkhart, 2015). Thus, it is suggested that the data derived from the present study and others like it be used by mixed, culturally competent committees, which can co-build the Strengthening Families Program for the Brazilian family context, respecting them in their values, necessities, and interests.

## Conclusions

In summary, this study provided evidence that SFP was perceived as sufficiently appealing, culturally relevant, and partially clear. Efforts for cultural adaptation should be targeted on linguistic aspects of the materials and procedures, considering the cultural, economical, and educational differences of the participant families, and carried on by culturally competent committees in a participatory way. Focus on implementation quality, including infrastructure, families’ mobilization, and continuous planning, should be prioritized. Lastly, a better understanding is needed for the impact of political context on the implementation process and the cultural adequacy of SFP for Brazilian families from non-urban areas, taking into account the social inequalities among Brazil’s regions and its geographic heterogeneity.

## Abbreviations

SFP 10-14-UK: Strengthening Families Program for children aged from 10 to 14, version adapted to the UK
SFP: Strengthening Families Program
